# Development and preliminary evaluation of Chinese School-aged Children’s Eating Behavior Scale

**DOI:** 10.1186/s41043-021-00265-8

**Published:** 2021-09-20

**Authors:** Hao Zhang, Xun Jiang, Yu-hai Zhang, Jing Yuan, Zhi-jun Tan, Tong Xu, Lei Shang

**Affiliations:** 1 Department of Health Statistics and the Ministry of Education Key Lab of Hazard Assessment and Control in Special Operational Environment, School of Public Health, Fourth Military Medical University, Xi’an, 710032 Shaanxi China; 2 Department of Pediatrics, Tangdu Hospital, Fourth Military Medical University, Xi’an, 710038 Shaanxi China

**Keywords:** Eating behavior, Scale, Reliability, Validity, School-aged children

## Abstract

**Background:**

The objective of this study was to develop a scale to assess eating behaviors of school-aged children (6–12 years old) in China.

**Methods:**

To develop the scale, a literature review and qualitative interviews were conducted. The draft scale contained 115 items and went through three evaluations among three groups of caregivers (*n* = 140, 400, 700) selected from suburban and urban kindergartens in Xi’an, Hanzhong, and Yanan, China, from March 2017 to October 2018. The psychometric properties of the scale were assessed using exploratory, confirmatory factor analysis, and variability analysis.

**Results:**

The final scale consisted of 46 items across eight dimensions including food fussiness, satiety responsiveness, food responsiveness, bad eating habits, susceptible diet, restrained eating, enjoyment of food, and junk food addiction. The total cumulative variance contribution rate was 52.16%. The scale and dimensions' Cronbach’s α coefficients, Guttman split-half reliability, and test- retest reliability were all above 0.65. The fitting indices for the confirmatory factor analysis were all close to 1. The scores for education of caregiver, family structure, and the body mass index of children were different among dimensions and groups, thus suggesting good discriminative utility.

**Conclusions:**

All of the results indicated that the scale has good reliability and construct validity for evaluating the eating behaviors of school-aged children in China.

## Background

The prevalence of overweight and obesity among children has been rapidly increasing globally in both developed and developing countries [1–3]. According to the standards of the Chinese Obesity Working Group (WGOC) in 2015, the prevalence of overweight and obesity in Chinese 7-year-old boys was 14.0% and 10.5%, respectively, and the corresponding prevalence in girls was 9.7% and 7.1% [4]. It is well known that overweight and obesity have substantial impacts on children’s health. Overweight and obese children are more likely to maintain the same adiposity levels throughout adolescence and adulthood [5, 6], and the adverse psychological and physical consequences of being overweight and obese in childhood may also persist into adulthood [7]. Therefore, childhood obesity has become a serious public health problem that requires urgent attention [8].

Obesity is a multifaceted disorder that results from the interaction of numerous factors [9]. Observational studies have evaluated behavioral risk factors and found that eating behaviors, measured by psychometric tools and parental report, play vital roles in developing childhood obesity [10–12]. Several instruments have been developed to assess children’s eating behaviors, such as the Children’s Eating Behavior Questionnaire (CEBQ) [13], the Dutch Eating Behavior Questionnaire for Children (DEBQ-C) [14, 15], among others [16–18]. All of these instruments have different structures, merits and applicability, and they may not work well in other populations due to vast variations in culture, ethnicity, and dietary habits. For example, CEBQ was developed in the United Kingdom (UK) and was translated and adapted into the Chinese version [19]; however, the dimensions in the Chinese version were not entirely the same as the original questionnaire. As an example, the factor ‘food responsiveness’ was divided into two items, while ‘enjoyment of food’ and ‘satiety responsiveness’ were not found.

Recently, children’s eating behaviors have received increased attention from both researchers and parents in China; however, most of the previous research includes epidemiological studies on the prevalence and factors of problematic eating behaviors [20–24]. In addition, most studies used self-developed non-validated questionnaires. Therefore, the assessment of eating behaviors may not be accurate and consistent across different studies. Substantial effort is needed to prevent overweight/obesity among children. Compared with other age groups, school-aged children (6–12 years) start to have increased self-consciousness, but poor self-control ability. Their learning ability and time spent in school also gradually increase, which provides an ideal opportunity to educate the children on healthy eating behaviors and prevent them from becoming overweight and obese. Due to the high overweight and obesity prevalence in China, it is important to have a valid and reliable instrument to be used by researchers and healthcare professionals to accurately measure the eating behaviors in Chinese school-aged children. However, to the best of our knowledge, no instrument has been validated and shown to be reliable for assessing school-aged children’s eating behaviors in China.

This study aimed to develop a scale (the Chinese School-aged Children’s Eating Behavior Scale [CSCEBS]) to objectively assess eating behaviors of school-aged children in China. The psychometric property of this scale was also evaluated.

## Methods

### Participants

**Fig. 1:**
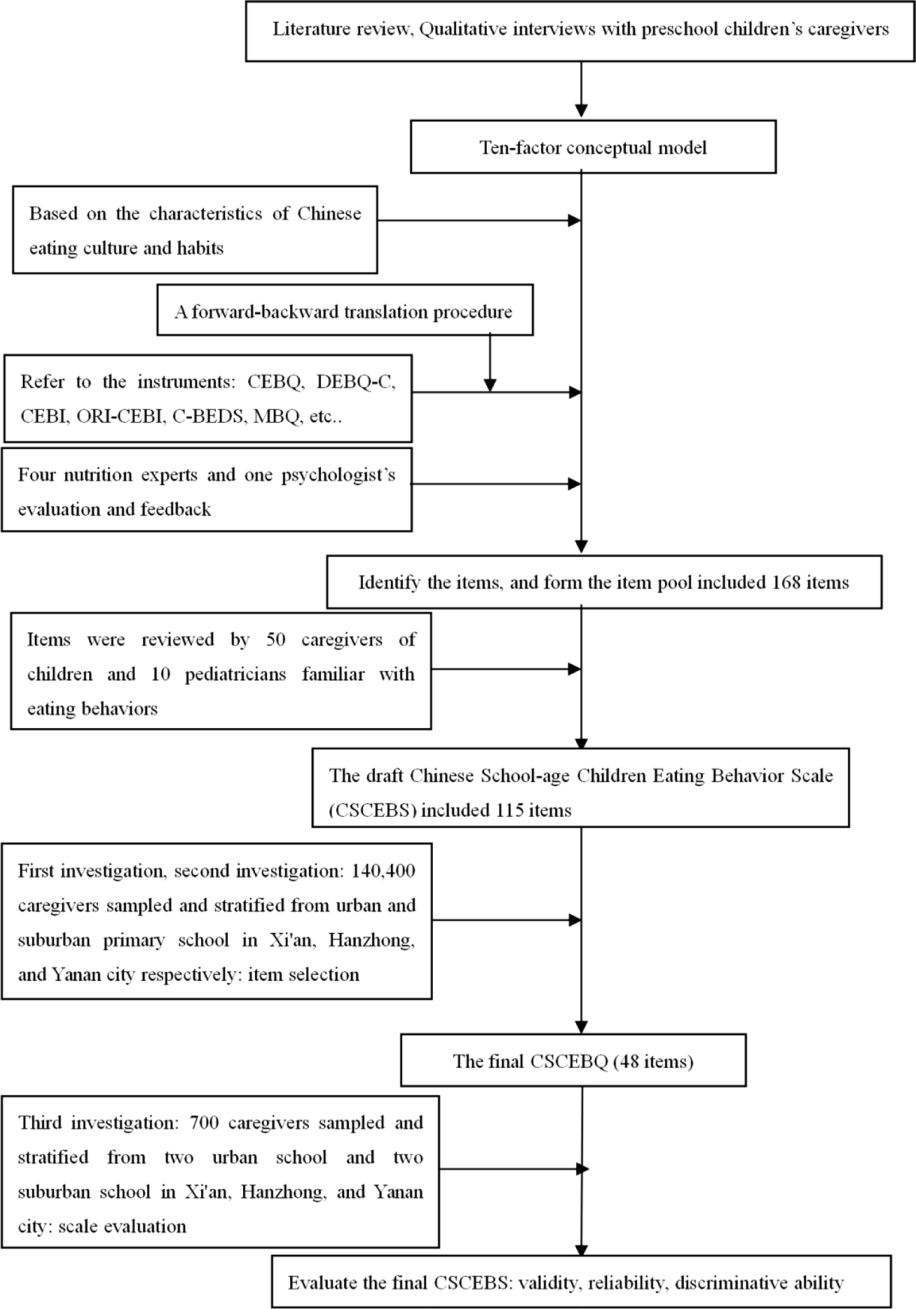
Flowchart of the research process and development and preliminary evaluation of the Chinese School-aged Children's Eating Behavior Scale

The present study was conducted in Xi'an, Hanzhong, and Yanan, China, between March 2017 and October 2018. To attain a representative population of school-aged children with difference in body weight, a random sampling technique was used to select schools located at different areas in these cities. The detailed study design is presented in Fig. 1.

Caregiver was defined as someone who takes care of a child on a daily basis. The current study used the following criteria to include participants: (1) the child was between 6 and 12 years old, (2) the child did not suffer from any disease that might influence the child’s appetite or eating behaviors in the past month, and (3) the caregiver of the child provided informed consent to take part in the survey. Caregivers who were uneducated or not willing to participate were excluded.

The Ethics Committee of the Fourth Military Medical University approved the current study. Written informed consent was provided by all recruited caregivers prior to the study, and the study complied with related regulations and guidelines. Data were collected anonymously.

Information on the age, sex, height, and weight of a child, and the caregiver’s levels of education, family structure, place of residence, and (family per capita) monthly income was collected using structured questionnaires. Eating behaviors of children using the scale developed in the current study, namely the Chinese School-aged Children’s Eating Behavior Scale (CSCEBS), were also evaluated. Prior to data collection, the aims, procedures, methods, each item’s meaning, and the significance of the study were explained, and instructions for filling out questionnaires were provided.

### Development of the Conceptual Model and the Draft Scale

Literature in English and Chinese published in the past 30 years was reviewed, two qualitative interviews were conducted, and a ten-factor conceptual model that systematically summarizes the eating behaviors of school-aged children (6–12 years old) was developed. During the first in-depth interview with 20 caregivers, information on school-aged children's eating behaviors to the greatest extent possible was collected using qualitative interviews. The eating behaviors from the first interview were then summarized and the information was used in the second interview. The second interview was conducted with 30 caregivers and was structured as a focus group discussion examining the generalizability of items in the outline. Results from the second interview were used to build the conceptual model that included ten dimensions: food fussiness, responsiveness to food, responsiveness to satiety, bad eating habits, external eating, emotional eating, independent and initiative eating, enjoyment of food, restrained eating, and junk food addiction.

Subsequently, 108 items were identified from the conceptual model and previous questionnaires (i.e., the DEBQ-C [14, 15], CEBQ [13], the Children Eating Behavior Inventory [16], the Oregon Research Institute Child Eating Behavior Inventory [17], Children’s Binge Eating Disorder Scale [25], Mealtime Behavior Questionnaire [26]). In addition, a third interview was conducted among six caregivers and three nutrition experts to combine the characteristics of Chinese eating culture, and 60 additional items were identified capturing the ten-factor conceptual model. A forward–backward procedure was used to translate items from existing instruments. A nutritionist and two bilingual professional translators conducted the forward (English–Chinese) and backward (Chinese–English) translation. One psychologist and four nutrition experts further evaluated the content validity (relevance, clarity, and ambiguity of items) of the Chinese scale version. As a result, an item pool of 168 items was formed.

Fifty caregivers of school-aged children and 10 experienced pediatricians reviewed the item pool and critically evaluated each item, including the importance of each item (correlations with eating behaviors) and the frequency. Each item ranged from 1 (not very frequent or important) to 5 (very frequent or important). Therefore, 60 scores for frequency and 60 scores for importance were scored for each item. A higher mean score of an item indicates increased importance or frequency. Items that had low frequency or importance (< 50th percentile) were further deleted. After the review, 55 items were removed due to low frequency or importance. Ultimately, a draft of the Chinese School-aged Children’ Eating Behavior Scale (CSCEBS) was created, which included 113 items.

### Methods for scoring

Each item of the scale measures the frequency of the corresponding eating behavior over the past two months. Five options were given for each item (“never,” “rarely,” “sometimes,” “often,” and “always”), and a corresponding number ranging from 1 to 5 was assigned respectively. Negative scores were given for reverse items. The mean score was calculated by dividing the total of all items by the number of items answered in each dimension. The score of the scale was the total of the scores in each dimension. A greater score for each dimension suggested a greater likelihood of children with this eating behavior.

### Investigation methods

The CSCEBS was administered to the main caregiver who had been feeding the child for more than 1 year and was very familiar with the child's daily diet. Five trained investigators were responsible for administering the questionnaire. The investigators first clarified the aim and process of the assessment, as well as the meaning of the questionnaire, to caregivers. Second, the height and weight of the child and the caregiver were measured using calibrated equipment (JT-918) by the investigators. Subsequently, caregivers completed the questionnaires to report their children’s eating behaviors over the past 2 months and returned the filled questionnaires to the investigators.

### First investigation: establishing the Trial Scale

The first investigation included 140 caregivers from two kindergartens in urban and suburban Xi'an using the stratified sampling methods. The first draft of the CSCEBS was completed by the caregivers independently and was used for analyzing items of the draft scale.

### Second investigation: constructing the Final Scale

The second investigation included 400 caregivers and utilized the same method and criteria from one urban and one suburban kindergarten in Xi’an, Hanzhong, and Yanan, respectively. The caregivers finished the trial scale of the CSCEBS independently for constructing the final CSCEBS.

### Third investigation: assessing the Final Scale

The third investigation included 700 caregivers from two suburban and three urban kindergartens in Xi’an, Hanzhong, and Yanan. The caregivers completed the final scale independently, and the dimensions of the scale and the reliability and validity were assessed. To test the test–retest reliability, 120 caregivers were randomly selected to complete the scale again after two weeks.

### Methods for quality control

The investigators carefully checked all questionnaires and conducted telephone interviews when spotting any missing information. Valid data from all completed questions were entered by using EpiData software. Double-entry and random check were used to ensure the data accuracy. SPSS was used to perform data analysis.

### Body Mass Index (BMI) classification

BMI (kg/m^2^) was calculated by measuring weight and height and was classified separately for children and caregivers. For children, overweight and obesity were defined using the Chinese guideline for children aged younger than 18 years of age [27]. Three groups were created: thinner weight (age- and sex-specified BMI < 10th percentile), normal weight (BMI ≥ 10th percentile to < 85th percentile), overweight/obesity (BMI ≥ 85th percentile).

### Data analysis

#### Item analysis

First, items that received the highest or lowest scores from over 15% of the caregivers indicated ceiling or floor effects, respectively [28], and were thus discarded. Second, the reverse scoring items were converted accordingly (5 = 1, 2 = 4, 4 = 2, and 1 = 5). Subsequently, five methods were used to select items [29]: the critical ratio analysis method, the discrete trend method, the correlation coefficient method [30], the exploratory factor analysis method [31], and the Cronbach’s α coefficient method [32]. The details of the five methods have been described previously [29]. Based on these methods, an item was deleted when it met exclusion criteria of three or more methods; if an item met the exclusion criteria of two methods, it was discussed with experts to decide whether it should be deleted or merged.

#### Reliability analysis

The Cronbach’s α coefficient, test–retest reliability coefficient, and split-half reliability coefficient were used for reliability testing. If coefficients were ≥ 0.70 and 0.60 [32] for total scale and dimensions, respectively, then the reliability was considered satisfactory.

#### Validity analysis

The samples were split into half and exploratory factor analysis and confirmatory factor analysis were used to explore and validate the structure [33]. A few fit indices were used to assess how well the model fit the data [31, 34]. The details have been described previously [31, 33–35]. In brief, the standardized root mean squared residual (*SRMR*) > 0.08, the adjusted goodness-of-fit index (*AGFI*) and the goodness-of-fit index (*GFI*) > 0.90, the comparative fit index (*CFI*) and the non-normed fit index (*NNFI*) > 0.95, the *χ*^2^/*df* < 5, and the root mean square error of approximation (*RMSEA*) < 0.05 indicated good model fit [31].

#### Discrimination analysis

The Student’s *t* test was used to compare scores of various dimensions between sex and place of residence. A one-way analysis of variance was used to compare scores by age, weight, education level of caregiver, monthly income, and family structure. SPSS was used to perform all statistical analyses. Continuous variables are presented as the mean ± standard deviation ($\overline{x} \pm s$), and categorical variables are expressed as frequencies and percentages. Two-sided *P* values < 0.05 indicate statistical significance.

## Results

### Participants’ characteristics

Characteristics of participants are presented in Table 1. A total of 140 caregivers were recruited for the first investigation (sample 1), and 115 (82.1%) questionnaires were valid. For the second investigation (sample 2), 400 caregivers were enrolled and 363 (90.8%) completed questionnaires. A total of 700 caregivers were recruited for the third investigation (sample 3) and 684 (97.7%) questionnaires were valid.

**Table 1:** Demographic information of Chinese school-aged children and their caregivers participating in the Chinese School-aged Children’s Eating Behavior Scale

Group	Sample 1 (*n* = 115)		Sample 2 (*n* = 363)		Sample 3 (*n* = 684)	
	*n*	%	*n*	%	*n*	%
*Gender*						
Boy	62	53.9	202	55.6	354	51.8
Girl	53	46.1	161	44.4	330	48.2
*Age (years)*						
6–7	26	22.6	110	30.4	151	22.1
8–10	51	44.4	130	35.8	298	43.6
11–12	38	33.0	123	33.8	235	34.3
*City*						
Xi’an	115	100.0	363	100.0	281	41.1
Hanzhong	–	–	–	–	257	37.6
Yanan	–	–	–	–	146	21.3
*BMI (kg/m*^*2*^*)*						
Thinner^a^	9	7.8	35	9.7	60	8.8
Normal^b^	95	82.6	284	78.2	531	77.6
Overweight/ obesity^c^	11	9.6	44	12.1	93	13.6
*Family structure*						
Nuclear family^d^	79	68.7	239	65.8	467	68.3
Stem family^e^	34	29.6	113	31.1	201	29.4
Single parent family	2	1.7	11	3.1	16	2.3
*Place of residence*						
Urban	83	72.2	201	55.4	370	54.1
Rural	32	27.8	162	44.6	314	45.9
*Education of caregiver*						
Junior high school or less	20	17.4	70	19.3	120	17.5
Senior high school	42	36.5	119	32.8	249	36.4
College or university or graduate	53	46.1	174	47.9	315	46.1
*Family per capita monthly income (RMB, Yuan)*						
< 3000	19	16.5	35	9.6	110	16.1
3000 ~	56	48.7	111	30.6	330	48.2
≥ 5000	40	34.8	217	60.6	244	36.7

^a^Age- and sex-specified BMI < 10th percentile

^b^Age- and sex-specified BMI ≥ 10th percentile to < 85th percentile

^c^Age- and sex-specified BMI ≥ 85th percentile

^d^Father, mother, and child

^e^Father, mother, grandparent, and child

### Item selection

From the first investigation, 31 items were deleted and 82 items were used to create a trial scale. Data of the second investigation were used to analyze and select items for the final scale. As a result, 36 items were deleted and a final scale was developed containing eight dimensions and 46 items. The detailed description for each item and the corresponding factor loading are shown in Table 2.

**Table 2:** Factor loading of the final Chinese School-aged Children’s Eating Behavior Scale (46 items) (*n* = 342)^a^

Dimension name and item	Loading	Dimension name and item	Loading
*Junk food preference, JP (19.49)*		24. My child would eat more when eating in a restaurant or other family	0.51
1.My child likes to eat fried food, such as chips and fried chicken	0.71	25. Even if the same food ingredients are made into different patterns or style, my children will eat more	0.51
2.My child likes to eat all kinds of puffed foods	0.68	26. My children eat more when there are guests at home	0.50
3.My child prefers eating instant noodles, hamburger, pizza and other fast foods over family meals	0.64	27. My child eats more and faster when happy	0.47
4.Between milk, yogurt and beverage, my child would choose beverage	0.64	28. My child chooses to eat when he/she feels lonely	0.46
5. My child especially likes to eat sweet foods, such as ice cream and candy	0.63	29. When someone plays with he/her, my child eats more	0.43
6.My child prefers to eat processed meat products, such as canned meat and sausage	0.62	*Restrained eating, RE (3.74)*	
7. My children like to eat food with heavy flavors, such as salty and spicy ones	0.50	30.During the meal, my child often says that if he/she eats too much, he/she will get fat	0.74
*Bad eating habit, BH (10.10)*		31. My child likes to eat meat, but he will limit the amount he eats at a time	0.74
8. During a meal, my child always plays while eating	0.65	32. My child often limits the amount of food he / she can eat for fear of gaining weight	0.70
9. Even if eats at home, my child doesn't want to serve foods for himself /herself	0.61	33. My child will limit himself/herself to drinks and snacks	0.65
10. If I do not allow to play with toys, watch TV or do other things, my child would not have his/her meal	0.54	*Enjoyment of food, EF (3.23)*	
11. During a meal, my child will eat more and more slowly	0.52	34. Whenever I give food, my child becomes happy	0.65
12. My child can sit before the dining-table obediently and finish his/her meal quickly^b^	0.52	35. My child would be happy when meal time comes	0.61
13. It takes more than 30 min for my child to eat his/her meal	0.50	36. My child is always asking for food	0.57
14. My child has leftovers at every meal	0.47	37. If allowed, my child will keep eating	0.56
15. My child often needs to be fed during a meal	0.46	38. My child is interested in food	0.46
*Food fussiness, FF (5.97)*		*Food responsiveness, FR (2.80)*	
16. My child only eats the food he/she selected	0.71	39. My child is always attracted to the food in the food shop	0.65
17. My child eats a limited variety of foods	0.67	40. My child wants to eat when watching others eat	0.64
18. My child doesn't eat foods he/she hasn't eaten before	0.63	41. My child wants to eat when smelling or seeing foods	0.60
19. My child refuses many foods because of the food’s taste, smell, appearance, texture, etc	0.59	42. Although my child is full, he/she could eat more when seeing his/her favorite foods	0.52
20. If there is a little food in the meals that he/she does not like, he/she would not eat	0.56	43. Whenever I give foods, my child would eat continuously	0.47
21. My child eats any kinds of foods^b^	0.48	*Appetite, AP (2.42)*	
*Susceptible eating, SE (4.41)*		44. My child eats less than other age-matched children	0.48
22. My child would eat more when using his/her favorite dishware	0.58	45. My child gets full up easily	0.46
23. My child eats more when there is nothing else to do	0.52	46. My child has a good appetite^b^	0.42

^a^This is half of Sample 3 sample size

^b^Items with reverse score

### Structure of the questionnaire

**Fig. 2:**
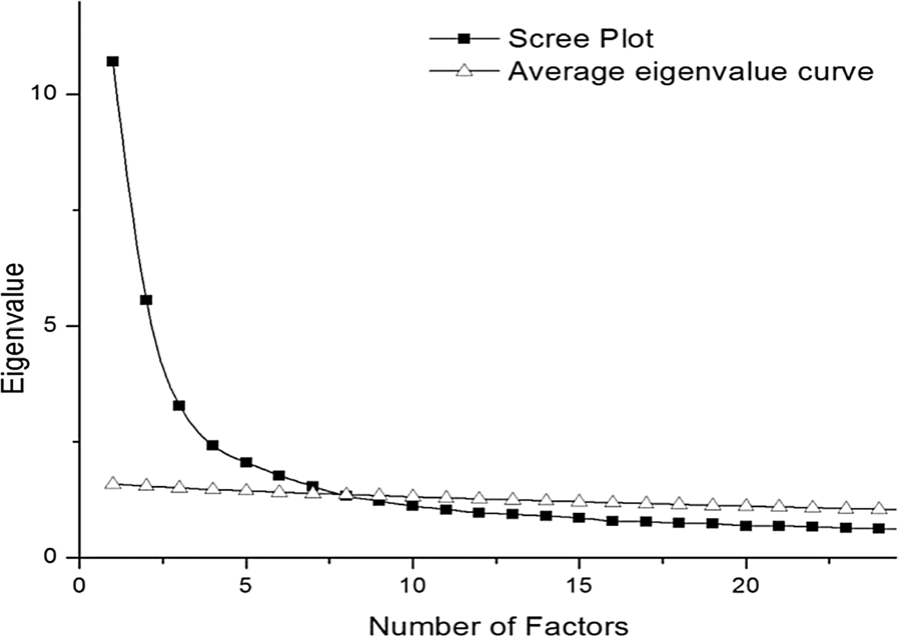
The parallel analysis plot shows the results from Scree plot and average Eigenvalue curve. The overlap of the two curves indicates the optimal factors that should be extracted. The plot indicates that eight factors should be extracted

The exploratory factor analysis was performed on half of the completed questionnaires randomly selected from the third investigation. The Kaiser–Meyer–Olkin of the sampling adequacy was 0.91 (> 0.6), and the Approx *χ*^2^ of Bartlett’s test of sphericity was 15,274 (*P* < 0.05). All results showed the fitness of data necessary for the exploratory factor analysis. The parallel analysis plot indicated that eight factors should be extracted (Fig. 2). The results of the exploratory factor analysis demonstrated that the Eigenvalues for these eight factors were 10.18, 5.55, 3.28, 2.42, 2.06, 1.78, 1.53, and 1.33, and the variance contribution rates were 19.49%, 10.10%, 5.97%, 4.41%, 3.74%, 3.23%, 2.80%, and 2.42%. The cumulative rate of variance contribution was 52.16%. The factor loadings of all items were above 0.4 (Table 2).

To define the possible implication of each factor, we summed up the implicit meaning of the item with the higher loading in each factor (Table 2). Factor one contained seven items and was named “junk food addiction (JA)”; it reflects a child’s preference for unhealthy food and drinks. Factor two included eight items named “bad eating habits (BH)”; it reflects a child's self-eating ability and unscientific, irregular, and unhealthy eating habits and behaviors. Factor three contained six items and was called “food fussiness (FF)”; it reflects a child’s desire to usually eat only a few foods of his/her choice and reject other foods. Factor four contained eight items and was named “susceptible diet (SD)”; it reflects that a child's eating speed and food intake amount are easily affected by external factors and their own emotions. Factor five contained four items and was called “restrained eating (RE)”; it reflects that a child deliberately controls the choice and amount of the food intake because he/she is worried about being overweight or obese. Factor six contained five items and was called “enjoyment of food (EF)”; it reflects a child’s enjoyment extent of all kinds of food. Factor seven contained five items and was named “food responsiveness (FR)”; it mainly reflects a child’s desire to eat after he/she sees, smells, or is served food. Factor eight included three items named “appetite (AP)”; it mainly reflects the amount of food and appetite of a child compared with children of the same age.

### Reliability

All 684 subjects from the third investigation were used to analyze the internal reliability of the scale. The Cronbach’s α coefficient was 0.89 for the total scale, and it ranged between 0.74 and 0.85 for the eight dimensions. The Guttman spilt-half reliability coefficient of the scale was 0.71, and it ranged between 0.65 and 0.81 for the eight dimensions. The two-week test–retest reliability coefficient of the scale was 0.73, and it ranged between 0.68 and 0.83 for the eight dimensions (Table 3). In sum, the results showed that the scale has good reliability.

**Table 3:** Reliability coefficients of the final Chinese School-aged Children’s Eating Behavior Scale in all dimensions (*n* = 684)

	Cronbach’s α coefficient	Guttman split-half reliability coefficient	Test–retest reliability coefficient
Junk food preference (JP)	0.85	0.78	0.83
Bad eating habits (BH)	0.82	0.81	0.75
Food fussiness (FF)	0.74	0.74	0.80
Susceptible eating (SE)	0.82	0.73	0.74
Restrained eating (RE)	0.84	0.77	0.78
Enjoyment of food (EF)	0.83	0.81	0.79
Food responsiveness (FR)	0.81	0.70	0.72
Appetite (AP)	0.79	0.65	0.68
Total	0.89	0.71	0.73

### Validity

The content validity ratio of the scale was 0.62. The mean time for finishing a survey was 23.0 ± 1.6 min, and participants said that they could easily understand the items of the scale. The correlation coefficient between scores of all dimensions ranged from 0.08 to 0.48; in comparison, the correlation coefficient between each dimension score and the total score was higher and ranged between 0.51 and 0.72. Confirmatory factor analysis was further used to evaluate the scale’s structure. Results of the statistics of the fit are shown as follows: *SRMR* = 0.05 (< 0.08), *χ*^2^/*df* = 1.92 (< 5), *GFI* = 0.90, *AGFI* = 0.89, *NNFI* = 0.92, and *CFI* = 0.93. Each index value was close to 1, and *RMSEA* = 0.04 (< 0.08). All of these indices met the statistical requirements [31, 35], indicating that the eight dimensions of the scale provided good fit for the data. Principal component analysis was conducted for each dimension of the scale, and only one factor had an Eigenvalue greater than 1 in each dimension. The variance contribution rate was between 45 and 71%, and the factor loading of each item in each dimension was greater than 0.4. Thus, the indices suggested an adequate structural validity of the scale.

### Discriminant ability

Table 4 shows that children in different age groups had significant differences in food fussiness, appetite, bad eating habits, susceptible eating, and restrained eating dimensions (*P* < 0.05). When age increased, the score of the dimensions of appetite and restrained eating also increased, and the score of dimensions of food fussiness, bad eating habits, and susceptible eating decreased. In addition, scores in food responsiveness, bad eating habits, and susceptible eating dimensions were significantly different among caregivers with different education levels (*P* < 0.05). Furthermore, scores in food responsiveness, food fussiness, bad eating habits, and restrained eating dimensions were different among children with variant family structures (*P* < 0.05). Scores were also different in various dimensions (except for food responsiveness and susceptible eating) among children with different body weights (*P* < 0.05). The scores of the enjoyment of food, appetite, food responsiveness, and junk food preference dimensions increased in higher weight categories, while food fussiness, bad eating habits, and restrained eating dimension scores decreased in higher weight categories. The scores were similar in all dimensions between men and women, among different places of residence, and with different family monthly incomes (*P* > 0.05).

**Table 4:** Comparison of each dimension score of the Chinese School-aged’ Children’s Eating Behavior Scale among different characteristics of children and caregivers in the third investigation of the scale ($\overline{x} \pm s$)

	*n*	Food fussiness	Food responsiveness	Appetite	Bad eating habit	Susceptible eating	Restrained eating	Enjoyment of food	Junk food preference
*Gender*									
Boys	354	2.49 ± 0.81	2.55 ± 0.77	2.36 ± 0.89	2.16 ± 0.71	2.23 ± 0.60	1.75 ± 0.81	3.26 ± 0.74	2.75 ± 0.80
Girls	330	2.52 ± 0.82	2.53 ± 0.79	2.36 ± 0.94	2.06 ± 0.65	2.25 ± 0.62	1.81 ± 0.78	3.26 ± 0.78	2.69 ± 0.76
*Age (years)*									
6–8	151	2.57 ± 0.83	2.54 ± 0.73	2.15 ± 0.78	2.17 ± 0.64	2.32 ± 0.60	1.72 ± 0.72	1.67 ± 0.73	2.76 ± 0.72
9–10	298	2.44 ± 0.82	2.48 ± 0.87	2.42 ± 1.02^a^	2.01 ± 0.65^a^	2.16 ± 0.64^a^	1.80 ± 0.80	1.81 ± 0.81	2.67 ± 0.85
11–12	235	2.40 ± 0.78^a^	2.44 ± 0.76	2.46 ± 0.92^a^	1.87 ± 0.59^a, b^	2.11 ± 0.57^a^	1.99 ± 0.89^a, b^	1.77 ± 0.80	2.72 ± 0.77
*Place of residence*									
Urban	370	2.51 ± 0.82	2.55 ± 0.79	2.36 ± 0.92	2.11 ± 0.68	2.25 ± 0.61	1.78 ± 0.80	3.27 ± 0.77	2.72 ± 0.78
Rural	314	2.45 ± 0.79	2.43 ± 0.69	2.33 ± 0.93	1.99 ± 0.66	2.19 ± 0.63	1.82 ± 0.75	3.21 ± 0.80	2.67 ± 0.74
*Education of caregiver*									
Junior high School or less	120	2.46 ± 0.74	2.42 ± 0.71	2.32 ± 0.92	2.05 ± 0.69	2.21 ± 0.56	1.83 ± 0.78	3.25 ± 0.68	2.80 ± 0.73
Senior high school	249	2.44 ± 0.85	2.48 ± 0.89	2.39 ± 0.99	2.02 ± 0.66	2.16 ± 0.64	1.84 ± 0.85	3.22 ± 0.80	2.70 ± 0.84
College or university or graduate	315	2.58 ± 0.79	2.67 ± 0.68^c, d^	2.35 ± 0.82	2.28 ± 0.67^c, d^	2.36 ± 0.59^d^	1.69 ± 0.75	3.20 ± 0.73	2.73 ± 0.71
*Family per capita monthly income(RMB, Yuan)*									
< 3000	110	2.36 ± 0.79	2.48 ± 0.85	2.36 ± 0.97	2.15 ± 0.68	2.17 ± 0.64	1.81 ± 0.76	3.19 ± 0.80	2.79 ± 0.79
3000 ~	330	2.49 ± 0.79	2.55 ± 0.80	2.43 ± 0.93	2.13 ± 0.68	2.27 ± 0.61	1.78 ± 0.82	3.26 ± 0.71	2.71 ± 0.77
≥ 5000	244	2.57 ± 0.84	2.55 ± 0.74	2.30 ± 0.87	2.07 ± 0.72	2.24 ± 0.61	1.79 ± 0.77	3.29 ± 0.80	2.71 ± 0.81
*Family structure*									
Nuclear family	406	2.45 ± 0.79	2.48 ± 0.75	2.36 ± 0.91	2.05 ± 0.66	2.21 ± 0.60	1.82 ± 0.81	3.24 ± 0.76	2.68 ± 0.77
Stem family	201	2.62 ± 0.87^e^	2.67 ± 0.82^e^	2.37 ± 0.92	2.25 ± 0.73^e^	2.31 ± 0.61	1.65 ± 0.76^e^	3.28 ± 0.77	2.82 ± 0.80
Single parent family	77	2.50 ± 0.75	2.58 ± 1.04	2.13 ± 0.93	2.23 ± 0.68	2.33 ± 0.82	1.82 ± 0.67	3.51 ± 0.80	2.55 ± 0.69
*BMI*									
Thinner	60	2.77 ± 0.80	2.25 ± 0.78	2.00 ± 0.82	2.41 ± 0.78	2.22 ± 0.56	2.01 ± 0.88	2.95 ± 0.74	2.26 ± 0.74
Normal	531	2.51 ± 0.81^f^	2.52 ± 0.77	2.34 ± 0.89^f^	2.29 ± 0.68^f^	2.24 ± 0.60	1.78 ± 0.79^f^	3.28 ± 0.74^f^	2.32 ± 0.78^f^
Overweight/obesity	93	2.22 ± 0.81^f, g^	2.70 ± 0.82^ g^	2.95 ± 0.97^f, g^	2.00 ± 0.61^f, g^	2.24 ± 0.76	1.49 ± 0.70^f, g^	3.39 ± 0.88^f^	2.79 ± 0.79^f, g^

^a^Compared with the age group of 6–8 years old *p* < 0.05

^b^Compared with the age group of 9–10 years old *p* < 0.05

^c^Compared with the group of junior high school and below *p* < 0.05

^d^Compared with the group of senior high school *p* < 0.05

^e^Compared with the group of nuclear family *p* < 0.05

^f^Compared with the group of thin *p* < 0.05

^g^Compared with the group of normal *p* < 0.05

## Discussion

The current study developed a new scale (CSCEBS) to evaluate the eating behaviors of Chinese school-aged children. The final scale consisted of 46 items across eight dimensions including food fussiness, satiety responsiveness, food responsiveness, bad eating habits, susceptible diet, restrained eating, enjoyment of food, and junk food addiction. CSCEBS showed good reliability and construct validity and could be useful to measure the complex eating behaviors of Chinese school-aged children.

It is very difficult to accurately measure eating behaviors in children because there are many factors that can affect the behavior. Although a few countries have developed some standardized tools to assess the eating behaviors of children, these scales were mainly developed in European countries and the United States (US), and thus no commonly recognized structure of questionnaires exists to evaluate the eating behaviors of children in China. In addition, these questionnaires or scales focused on children in different age groups and with varying cultural backgrounds. For instance, the DEBQ-C was developed for Dutch children aged 7–12 years old [14, 15], and the CEBQ was designed for UK children aged 2–9 years old [13]. Similar to the CEBQ-C, the new scale presented here included dimensions of responsiveness to food, enjoyment of food, food fussiness, and restrained eating; different from the CEBQ-C, this new scale also included 5 other dimensions. A few dimensions from the CEBQ-C (desire to drink, slowness in eating, emotional eating, satiety responsiveness, exogenous eating, and susceptible eating) were not included as a single dimension in the current study; instead, they were incorporated with other items as a new dimension (junk food addiction, bad eating habits, susceptible eating, and appetite). Compared with DEBQ-C, the dimension of restrained eating was also extracted in our scale, exogenous eating was not extracted alone, but the dimension of susceptible eating included 5 items reflecting exogenous eating. Furthermore, the Chinese Preschool Children Eating Behavior Questionnaire (CPEBQ) was developed for preschool children aged 3–6 years old [18], and consisted of 38 items and seven dimensions (food responsiveness, food fussiness, satiety responsiveness, eating habits, emotional eating, exogenous eating, and initiative eating). Compared to the CPEBQ, the CSCEBS has more dimensions and items. All of these differences among scales may be due to the different eating behaviors of children from different countries, regions, and age groups. Therefore, further studies are warranted to validate our scale among children in the same age group and cultural background.

The development of food preferences in children involves a complicated interaction of genetic, family, and environmental factors [36]. Although recent studies suggest that genetic influence could be a strong determinant of appetite in children, environmental factors are also important in determining eating behaviors of children. After assessing the factors associated with children's eating behavior, Scaglioni et al. [36] concluded that children's eating behaviors are influenced by genetics, family environment, social environment, and other factors. Another study evaluating the eating behaviors of Chinese children aged 6–12 years old reported that sex, race, weight, and the educational levels of parents are associated with the eating behaviors of children [12]. Consistent with these two studies, the current study found significant variations in dimension scores among different education levels of caregivers, family structure, and children's weight [12, 36]. In addition, different eating behaviors have been found between adolescent boys and girls, but less is known about the starting age of gender difference [37]. Consistent with Wardle [37], the results of this study showed that scores in all dimensions were similar between boys and girls, although these findings are partially different from those of Webber [12]. Among the eight dimensions in the present scale, the scores for food fussiness, susceptible eating, and bad eating habits were inversely associated with age, and the score of restrained eating and appetite were positively associated with age. The findings of this study are consistent with a UK study using the CEBQ [38], which indicated that eating behavior has attributes of stability and continuity in children. In addition, Nakao et al. [39] showed that the weight of a child correlates with his or her eating behavior at age 4 years. The present study showed that the scores of food fussiness, appetite, bad eating habits, restrained eating, food responsiveness, junk food preference, and enjoyment of food were significantly different among children with different BMI categories (thinner, normal weight, and overweight/obese), which corroborated previous findings that eating behavior is the key factor of childhood overweight and obesity [12]. However, different from previous studies [18, 20–24, 36], the score of each dimension did not differ by family income and places of residence. The heterogeneity may be due to the fact that the subjects recruited in the current study were from Shaanxi province, where the variations in economic levels among different regions, and between urban and rural areas, were smaller compared to previous studies.

Previous studies assessing eating behaviors among Chinese school-aged children have found that common eating behaviors among this population include food fussiness (26.7–77.3%), eating slow (43.3–46.4%), eating less (18.3–41.3%), food refusal (30.0–47.8%), unwilling to experiment with new foods (17.6–33.9%), favor foods with special type, color, texture, etc. (26.7–71.6%), no fixed site for eating (30.0–45.8%), multi-task while eating (32.2–60.1%), eating influenced by external factors (13.7–36.9%), emotional eating (8.6–16.8%), bad eating habits (54.2%), and eating high calorie food, high sugar food, etc. (47–70.0%) [20–24]. The eight dimensions of the CSCEBS developed in the current study (food fussiness, food responsiveness, satiety responsiveness, bad eating habits, susceptible diet, restrained eating, enjoyment of food, and junk food addiction) covered all the above-mentioned common eating behaviors among school-aged children in China. Therefore, the results presented here highlight that the CSCEBS is suitable for assessing the eating behaviors among this population.

This study has important clinical implications. Evaluating eating behaviors is crucially informative for developing intervention programs to provide education for children with problematic eating behaviors, as well as their families. A higher dimension score suggests that the child is more likely to have problematic eating behavior in this dimension. The CSCEBS is a useful and convenient tool and could be widely applied to all hospitals, healthcare centers, and schools in China. Using the CSCEBS, childcare workers or pediatricians could quickly identify eating problems and develop targeted interventions and support for children and their families.

The current study has some strengths that should be noted. The CSCEBS was developed by integrating information from other Chinese, European, and US scales, and effectively combined overlapping and different dimensions of content from these scales. Comprehensive statistical analyses were used to develop and evaluate the psychometric properties of the scale. For example, both exploratory and confirmed factor analyses were performed, and a few dimensions from previous studies were grouped [31–33] into one dimension in the current study, which made the scale more representative and targeted for the current population. However, several limitations of this new scale merit consideration. First, children’s eating behaviors varied between regions and were multifactorial, with distinct differences across locations and ethnicities [10, 36, 40, 41]. Since the current study was conducted in Shaanxi Province, it is possible that the scale did not capture eating behaviors that are unique in other regions of China. Second, participants were selected from three cities (Xi’an, Hanzhong, and Yanan) and therefore the findings might not represent other cities in the same province or other provinces in China. Furthermore, since there is no “gold standard” tool or a generally accepted tool to measure school-aged children's eating behaviors in China, the criterion validity was not assessed. Given that eating behaviors may vary with cultural background and that the prevalence of childhood obesity has been increasing in China, validation studies among other populations are vital to confirm our findings. Future studies are warranted to confirm the reliability and validity of this scale in other geographical regions and ethnic groups in China.

## Conclusions

This study presents a new scale to measure the eating behaviors among school-aged children in China. The scale is a theory- and evidence-based tool for assessing school-aged children's eating behaviors. This scale was reliable and valid with good discriminative ability in a population of school-aged children in China. Future studies should confirm existing findings in different Chinese populations with larger sample sizes. Additional studies are needed to explore how eating behavior influences children’s weight and identify effective strategies to prevent childhood obesity.

## Data Availability

The datasets used and analyzed during the current study are available from the corresponding author on reasonable request.

## Abbreviations

WGOC: Chinese Obesity Working Group
CEBQ: Children’s Eating Behavior Questionnaire
DEBQ-C: Dutch Eating Behavior Questionnaire for Children
CSCEBS: Chinese School-aged Children’s Eating Behavior Scale
CPEBQ: Chinese Preschool Children’s Eating Behavior Questionnaire
*SRMR*: Standardized root mean squared residual
*AGFI*: Adjusted goodness-of-fit index
*GFI*: The goodness-of-fit index
*CFI*: Comparative fit index
*NNFI*: Non-normed fit index
*RMSEA*: Root mean square error of approximation
JA: Junk food addiction
BH: Bad eating habits
FF: Food fussiness
SD: Susceptible diet
RE: Restrained eating
EF: Enjoyment of food
FR: Food responsiveness
AP: Appetite
